# Identification of Serum Peptidome Signatures of Non-Small Cell Lung Cancer

**DOI:** 10.3390/ijms17040410

**Published:** 2016-03-31

**Authors:** Agnieszka Klupczynska, Agata Swiatly, Joanna Hajduk, Jan Matysiak, Wojciech Dyszkiewicz, Krystian Pawlak, Zenon J. Kokot

**Affiliations:** 1 Department of Inorganic and Analytical Chemistry, Poznan University of Medical Sciences, Grunwaldzka 6 Street, 60-780 Poznan, Poland; a.klupczynska@gmail.com (A.K.); agata_swiatly@wp.pl (A.S.); jo.hajduk@gmail.com (J.H.); jmatysiak@ump.edu.pl (J.M.); 2 Department of Thoracic Surgery, Poznan University of Medical Sciences, Szamarzewskiego 62 Street, 60-569 Poznan, Poland; dyszkiewicz@wp.pl (W.D.); krystianp@hotmail.com (K.P.)

**Keywords:** peptide profiling, non-small cell lung cancer, MALDI-TOF-MS, ZipTip enrichment

## Abstract

Due to high mortality rates of lung cancer, there is a need for identification of new, clinically useful markers, which improve detection of this tumor in early stage of disease. In the current study, serum peptide profiling was evaluated as a diagnostic tool for non-small cell lung cancer patients. The combination of the ZipTip technology with matrix-assisted laser desorption/ionization time-of-flight mass spectrometry (MALDI-TOF-MS) for the analysis of peptide pattern of cancer patients (*n* = 153) and control subjects (*n* = 63) was presented for the first time. Based on the observed significant differences between cancer patients and control subjects, the classification model was created, which allowed for accurate group discrimination. The model turned out to be robust enough to discriminate a new validation set of samples with satisfactory sensitivity and specificity. Two peptides from the diagnostic pattern for non-small cell lung cancer (NSCLC) were identified as fragments of C3 and fibrinogen α chain. Since ELISA test did not confirm significant differences in the expression of complement component C3, further study will involve a quantitative approach to prove clinical utility of the other proteins from the proposed multi-peptide cancer signature.

## 1. Introduction

Lung cancer (LC) has been the leading cause of malignant tumor-related deaths for years [1,2]. Due to poor clinical manifestation and low effectiveness of diagnostic tools, LC is usually diagnosed at an advanced stage, which results in the highest mortality rates among all cancers, in both men and women. The prognosis of LC is generally poor and the overall five-year survival rate reaches only 15% [3]. Currently, the LC diagnosis is mainly based on the CT or PET/CT of the chest. A low-dose computed tomography was suggested as an additional screening tool, that can contribute to early LC detection. However, that technique is limited by a low specificity resulting in a large number of false positives [3,4,5].

Therefore, there is an urgent need for identification of new, clinically useful biological markers of LC that can enhance the current diagnostic capabilities. Biomarkers that can be used in a minimally invasive screening test would complement diagnostic imaging and have an immediate impact on better survival in LC patients. To identify potential LC biomarkers different strategies have been used. An increasing interest in searching for biomarkers of LC occurred in two rapidly expanding fields of science—proteomics and metabolomics. Both of them use different mass spectrometry techniques as an analytical tool for searching abnormalities in proteome and metabolome of LC patients. A number of global metabolic profiling studies of non-small cell lung cancer (NSCLC) patients using gas chromatography-mass spectrometry [6,7,8,9,10] or liquid-chromatography-mass spectrometry [7,9,11,12] was presented. Mass spectrometry-based targeted metabolomic approach revealed altered levels of some plasma free amino acids in NSCLC subjects [13,14]. Another promising approach in discovery of candidate LC biomarkers represents proteomic research. The identification of differences in proteome between healthy and diseased individuals is a major aim of clinical proteomics [15,16]. Several proteins were proposed as the potential LC markers, among which the most extensively investigated are cytokeratin 19-fragments (CYFRA 21-1), neuron-specific enolase (NSE), carcinoembryonic antigen (CEA) and squamous cell carcinoma antigen (SCC) [17,18]. However, their clinical applicability in LC detection is limited. Although the concentration levels of CYFRA 21-1, CEA and SCC change along with the development of LC, they are characterized by insufficient sensitivity and specificity to be used as stand-alone diagnostic tests, whereas NSE is a useful prognostic factor only in the advanced stages of the disease [4,19]. Mass spectrometry was found to be the most powerful tool among the proteomic technologies. An overview of research focused on discovery of new diagnostic and prognostic markers of LC through mass spectrometry-based proteomic analyses has been published recently [20]. In searching for a noninvasive diagnostic tools in LC detection breath analysis was also tested. Among volatile organic compounds present in exhaled breath alcohols, aldehydes, ketones and hydrocarbons were found to be potential markers of LC [21]. Despite the encouraging data emerged from several studies none of the proposed biomarkers has reached clinical utility so far [22,23,24].

In proteomic and metabolomic research and the analysis of exhaled breath it was indicated that a panel of biomarkers has a higher discriminatory power in LC detection than a single compound alone [13,24,25,26]. The use of a single compound for cancer diagnosis has a higher risk of producing false-positive results caused by links with other disorders. Due to heterogeneity of LC a panel of markers seems to be more appropriate from the clinical point of view [27]. Therefore, it can be supposed that the study of a pattern of multiple compounds can lead to obtain many important diagnostic information simultaneously and provide enhanced sensitivity and specificity in patients’ classification.

In searching for new cancer biomarkers, the expression pattern of multiple proteins has been investigated using diverse methods, such as an antibody microarray [28], surface-enhanced laser desorption/ionization time-of-flight mass spectrometry (SELDI-TOF-MS) [25] and matrix-assisted laser desorption/ionization time-of-flight mass spectrometry (MALDI-TOF-MS) [29,30,31]. The use of MALDI-TOF-MS profiling enables to obtain a whole pattern of low-molecular weight proteins occurring in human serum and was indicated as a promising strategy in several cancer research. This technology was found to be effective in discrimination of healthy individuals from patients with various tumors such as biliary tract cancer [32], gastric cancer [33], prostate cancer [34], ovarian cancer [16] and head and neck cancer [35]. Moreover, serum peptidome features were suggested to be useful in monitoring the toxicity of applied therapy in women with breast cancer [36].

Although the use of serum peptide profiling holds promise in diagnostics, there are several debate topics related to this approach. One of the most commonly discussed concern is a lack of reproducibility of obtained data across different published studies. It should be bear in mind that cancer-related biomarkers occur in blood at very low concentration levels in comparison to proteins such as albumin and immunoglobulins, which mask other compounds. Apart from high-abundant proteins, the presence of lipids and salts can also adversely affect the peptide pattern profiling [37,38,39]. A range of sample preparation techniques was developed to remove the abundant proteins and desalt samples. One of the most widely used depletion techniques in MALDI profiling studies is solid phase extraction. Taking into account that reproducibility of the MS profiling is highly dependent on sample preparation procedure in our study solid phase extraction using C18-ZipTip pipette tips based on reversed-phase chromatography was chosen as a sample pre-treatment method. To the best of our knowledge, this is the first research that presents the application of ZipTip technology for purifying the serum samples of LC patients. In this technique, the molecules are separated due to an adsorptive process based on their chemical properties and hydrophobic characteristics. The separation is determined by hydrophobic interaction between the analytes in the mobile liquid phase and a solid stationary phase with immobilized hydrophobic ligand [40]. The ZipTip enrichment strategy was proved to be a reliable method for sample preparation in MS peptide profiling [38,39].

The goal of the present study was to determine serum peptide profiles of patients with NSCLC and matched healthy controls by MALDI-TOF-MS technology combined with C18-ZipTip packed tips. Patients with early stage NSCLC represented a significant part of the studied group, which is particularly valuable in terms of searching for early cancer biomarkers. The classification model obtained based on peptide pattern was verified using a validation set of samples. The selected discriminating peptide peaks were then identified using liquid chromatography-tandem mass spectrometry.

## 2. Results

### 2.1. Characteristics of the Study Participants

In the current study, serum peptide pattern profiling was evaluated as a diagnostic approach for NSCLC patients. The characteristics of cancer patients and the control group are summarized in Table 1. Histologically, 55.6% of tumors (*n* = 50) were diagnosed as squamous cell carcinomas and 44.4% of them (*n* = 40) as adenocarcinomas. The most often diagnosed grade of cancer differentiation was G2 (52.0%). According to TNM Classification for Lung Cancer the most common stages in the study group were as follows: IB (26.0%), IIA (26.0%) and IA (20.0%). Thus, patients with early stage NSCLC represented a significant part of the studied group. None of the patients had diagnosed NSCLC at IV stage. The study group and control group had similar percent of men and women. No statistically significant differences in age occurred between LC patients and controls (*p* = 0.2378).

### 2.2. Reproducibility Evaluation

Both application of ZipTips for sample pretreatment and the serum peptide profiling were proved to be reliable and reproducible technologies [38,39]. Nevertheless, we performed some additional experiments in order to check reproducibility of the whole applied procedure in our experimental conditions and to confirm that the variation in spectra shows biological differences in intensities of peptide ions rather than systematic variability. The intra-day reproducibility of the protein profiles was evaluated by analyzing samples spotted at three different target positions. Variability assessment was based on eight randomly selected peaks with low, medium and high peak area in a mass range of *m*/*z* 1–10 kDa. The coefficient of variation (CV) calculated for intra-day reproducibility varied from 2% to 10% (average CV = 6.9%) (Table S1). The inter-day reproducibility was evaluated using spectra obtained from the same samples but on three different days. The CV values ranged between 2% and 42% with an average inter-day variability of 20% (Table S2). It can be concluded that both intra-day and inter-day studies proved that the applied methodology yields a high quality results and is suitable for searching for cancer-related differences at peptidome level.

### 2.3. Serum Peptide Profiling

The current research involved the application of peptide profiling in serum samples collected from patients with diagnosed NSCLC and the matched control group. In total, 153 serum samples derived from cancer patients (*n* = 90) and healthy controls (*n* = 63) were subjected to C18 reversed-phase extraction using ZipTips and analyzed by MALDI-TOF-MS. The performed analyses allowed to detect 136 unique peaks. Univariate statistical analyses allowed to find peptides which were significantly different between the analyzed groups (Table 2). Their diagnostic efficiency was further examined by receiver operating characteristic (ROC) curve-based analyses. The area under the ROC curve (AUC) values above 0.75 confirmed high accuracy of peptide ions in discrimination between NSCLC patients and controls without cancer. Eight peptides of 1520.16, 1546.72, 1568.45, 1617.88, 2083.30, 4466.98, 4787.36, and 4803.17 Da had the most discriminative power with *p*-value < 0.00004 and AUC values ranging from 0.75 to 0.85 (Table 2).

The serum peptide patterns were also subjected to multivariate statistical analysis, which considers multiple variables simultaneously and takes into account correlations between variables. The classifications of the samples were tested using three different algorithms: genetic algorithm (GA), quick classifier (QC) and supervised neural network (SNN). The discrimination models were generated based on a training set containing the randomly assigned 67 NSCLC samples and 47 control samples. Two indicators of the model’s performance, cross validation and recognition capability, were calculated (Table 3).

As can be seen in Table 3, among the three classification algorithms, the GA-based model showed the highest values of cross validation (71.89%) and recognition capability (96.22%). Due to the best efficiency in the discrimination between NSCLC group and the control group further statistical analyses were performed using only GA model. The GA-based classification model was composed of 10 peptides including four peaks selected earlier by univariate statistical tests (1520.16, 1546.72, 1568.45, and 1617.88 Da) (Table 3). The poor discriminatory ability of SNN model can result due to small number of peptide ions included into the model. In order to verify the classification capabilities of GA model, external validation was performed using a test set (Table 1). Based on analysis of new serum samples, it was indicated that the generated model discriminated the two studied classes with a sensitivity of 64.40% and a specificity of 94.40% (Table 3).

Additional statistical analyses were carried out for two main histological types of NSCLC, adenocarcinomas and squamous cell carcinomas separately. Characteristics of the classification models generated with regard to NSCLC subtypes are presented in Table 3. Despite some discrepancies existing between the generated models, it can be noticed that peptides of 1568.45 and 1450.94 Da were associated with adenocarcinomas, while peptide of 1520.16 Da was related to squamous cell carcinomas. Peptide of 1546.72 Da was found to have a high discriminative ability regardless the histological type of NSCLC.

### 2.4. Identification of Peptide Ions

**Figure 1:**
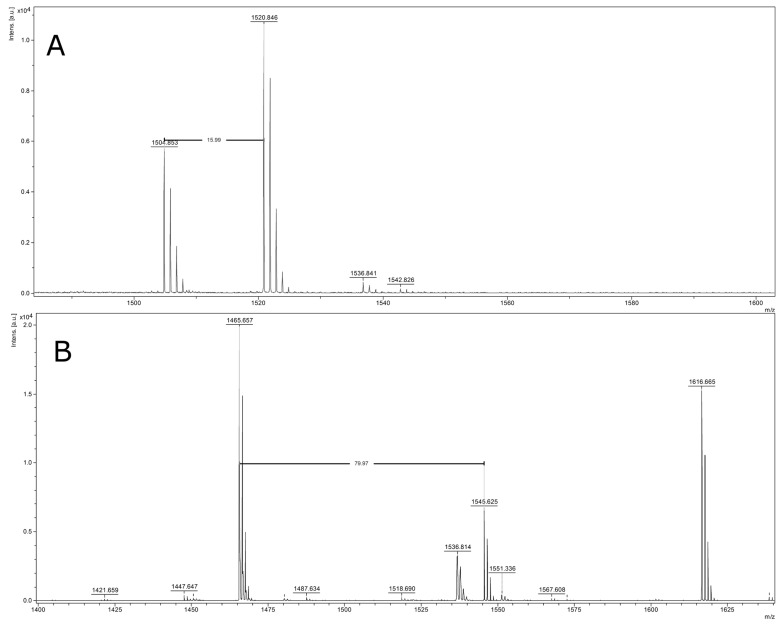
(**A**) Mass spectrum of *m*/*z* 1520.8456 with assigned oxidative modification of *m*/*z* signal 1504.853; (**B**) Mass spectrum of *m*/*z* 1545.6249 represent with assigned phosphorylation modification of *m*/*z* signal 1465.657.

The conducted statistical analyses allowed to select some ions from the whole peptide pattern occurring in human serum, which had the highest discriminatory abilities in LC detection. In order to identify chosen peptide ions (linear positive mode *m*/*z* 1520.16, 1546.72, 1568.45, and 1617.88 Da) additional experiments were carried out using LC-MALDI-TOF/TOF-MS/MS technique. Spectra were automatically recorded in the reflector mode in the mass range 700 to 3500 Da. As many peaks detected in the low mass range were presented in the close neighborhood, the identification of the chosen peaks by MALDI-TOF/TOF method seemed to be challenging. For this reason, we decided to extend the time of nLC gradient from our previous method [41]. This procedure allowed to obtain effective peptide separation and detection of all discriminative peaks. The MS analysis of signal *m*/*z* 1520.8456 showed the oxidation modification (16 Da) of signal 1504.8527, which is presented on the Figure 1A. The MS/MS fragmentation of precursor ion *m*/*z* 1520.8456 allow to identify complement C3 (CO3_HUMAN) protein with the peptide sequence of SPMYSIITPNILR with a significant score in the Mascot search. The MS/MS spectrum of peptide ion *m*/*z* 1520.8456 Da is shown in Figure S1.

The MS/MS analysis of signal *m*/*z* 1545.6249 resulted in identification of the following peptide sequence: DSGEGDFLAEGGGVR (Figure S2). The interpretation of MS spectrum of the peak 1545.6249 revealed the presence of phosphorylation modification (79.98 Da) of the *m*/*z* 1465.657 (Figure 1B). The matched sequence gave a significant score in the protein database to fibrinogen α chain (FIBA_HUMAN) protein. Analysis of *m*/*z* 1568.67 and 1616.67 failed, as the contamination of other neighborhood fragments were observed in the MS/MS spectrum. For this reason, *m*/*z* 1568.67 precursor with hypothetical peptide sequence DSCSRDGALLGCSLTA did not give any reliable identification. In the MS-BLAST search homology to Uncharacterized family 31 glucosidase KIAA1161 was found. The MS/MS fragmentation of *m*/*z* 1616.67 led to obtain AEIQGKMEDLPEQE peptide sequence. The structure contains additional unknown modifications, which may hinder its identification. Thus, the established sequence resulted with no significant hit in Mascot search. Therefore, further study involving the identification of both peptide ions of 1568.67 and 1616.67 Da should be done.

Validation of candidate protein markers identified based on MALDI-TOF-MS analysis should be performed using quantitative approaches such as an antibody-based technology or a mass spectrometry-based method. An enzyme-linked immunosorbent assay (ELISA) was used for verification of differences in the expression of complement component C3 in the analyzed serum samples. Despite in NSCLC group elevated concentration of complement C3 was observed compared to the control group (0.17 *vs.* 0.16 g/L) the difference was not statistically significant. Since ELISA test for determination of fibrinogen α chain in serum is not commercially available, future study will involve development of multiple reaction monitoring (MRM) method using triple quadrupole mass spectrometry, in which targeted measurements of the selected peptides are performed. Moreover, the MRM-based approach seems to be more reliable, because difference in the intensity of peptide ion derived from fibrinogen α chain can be also caused by cancer-specific exopeptidases.

## 3. Discussion

Despite a similar incidence to other malignant tumors, such as breast and prostate cancer, LC causes 4–5 times more deaths [2]. This discrepancy is partly associated with the fact that there are no satisfactory early diagnostic strategies for detection of lung malignant tumors. Hence, there is a need for identification of new, clinically useful markers, which improve detection of LC in its early stages. Identification of detectable abnormalities in low-molecular weight serum proteome is one of the strategies, which are now under intensive investigation. The aim of the study was to search for serum peptidome signatures of NSCLC patients and to assess their utility in the detection of that tumor. MALDI-TOF-MS is a high throughput technology that provide an opportunity to obtain polypeptide profiles which, combined with sophisticated bioinformatic tools, have a great impact on the acceleration of the work aimed at development of new diagnostic tests for cancer.

The combination of the ZipTip enrichment with MALDI-TOF-MS for the analysis of serum peptide spectra of LC patients was reported for the first time. There are few reports in the available literature, which present MALDI-TOF-MS analyses of blood proteome profiling in LC, however all of them are based on the usage of magnetic bead-based technologies for sample pretreatment [29,30,31]. It was indicated that the sample preparation procedure has a great influence on the obtained protein profiles [39]. Since the sample preparation method is an important issue and determines the success of the whole experiments, it also affects the discriminatory abilities of the peptide pattern in detection of cancer and other diseases. Consequently, the peptide ions proposed as candidate LC biomarkers are different when compare various studies. The discrepancies in the discriminatory peptides occurring in serum of LC patients can result not only from diverse analytical methodologies, but also from different histological type and clinical stage of cancer. In the presented study, 46.7% of the patients had NSCLC at stage I, whereas in the study of Musharraf *et al.* [31] subjects at stage III and IV were mainly analyzed. It can be supposed that patients with stage I represent more valuable group from the viewpoint of the development of a test for early LC detection. Shevchenko *et al.* [29] and Lin *et al.* [30] analyzed only one subtype of NSCLC, squamous cell carcinomas and adenocarcinomas, respectively. In our research, these two most common NSCLC subtypes were involved and thus the findings may refer to a wider group of patients.

Based on our research, a number of low-molecular weight proteins were found to be differentially expressed in the serum samples of NSCLC patients compared to the control subjects. Some of the peptides were identified as discriminative based on both univariate and multivariate (GA) statistical analyses. These peptides include: 1520.16, 1546.72, 1568.45, and 1617.88 Da. As can be seen in Table 3, different algorithms used for generation of the classification models resulted in different peak selection. A lack of complete consistency in terms of peak selection to the generated models was not surprising, because the applied algorithms used various inclusion criteria for peptide ions, such as univariate sorting or prototype-based classification. Despite the apparent differences concerning peptide selection to the models, some shared traits between the generated models can be noticed. Two peptide ions of 1546.72 and 1617.88 Da were common to all three applied algorithms, which indicates their potential as NSCLC candidate biomarkers (Table 3). Moreover, some discrepancies in the results of multivariate analyses (GA, QC, and SNN) between two the most common histological types of NSCLC also existed. Nevertheless, it was found that peptides of 1568.45 and 1450.94 Da significantly correlated with adenocarcinomas, whereas peptide of 1520.16 Da was characteristic feature of squamous cell carcinomas. Based on Table 3, it can be concluded that peptide of 1546.72 Da had a high classification ability regardless the histological type of NSCLC. Our study confirmed the hypothesis that due to heterogeneity of NSCLC a panel of markers will have better performance in NSCLC detection than a single compound. It is worth noting that GA-based model for discrimination between NSCLC patients and controls consisted of peptides identified as characteristic traits of both adenocarcinomas and squamous cell carcinomas. This could explain the highest classification abilities of that model.

External validation was conducted to assess robustness of the generated classification models. This is an important step in data analysis, which enabled verification of the obtained preliminary results and discrimination of false positives from markers of high potential in clinical practice. The analysis of a new validation set of serum samples revealed satisfactory values of sensitivity and specificity for GA-based model (94.40% and 64.40%, respectively), proving the reliability of the model. Thus, only a combination of 10 peptide ions was robust enough to detect new set of NSCLC samples. Six of them were observed at higher intensity levels in NSCLC group (1466.90, 1568.45, 1617.88, 1520.16, 1546.72, and 5904.39 Da) and four of them were found significantly decreased in cancer patients (1450.94, 1880.97, 6330.86, and 6432.69 Da). This finding once more supports the assumption that a serum multi-peptide signature could be applied for detection of patients with NSCLC. The results of our studies should be confirmed by an analysis of additional set of serum samples. The sample size involved in the external validation procedure was 39, therefore further studies with greater number of samples should verify the obtained promising findings. Due to the fact that many serum proteins can be related to inflammatory processes, future studies should also include a set of samples collected from patients with inflammatory conditions as an additional control group.

Peptides of the highest discriminatory ability were subjected to further experiments with the aim of their identification as fragments of specific proteins. The LC-MS/MS analysis of *m*/*z* 1520.8456 peak led to identification of complement C3 (CO3_HUMAN) protein with the peptide sequence of SPMYSIITPNILR. In the current study, higher intensity of this peptide was found in NSCLC group. Complement C3 plays a key role in the activation of the complement system, which is an essential part of immune system and regulates many immunological and inflammatory processes. The interactions between complement and cancer turned out to be more complex than expected [42,43]. A number of studies indicate that cancer cells activate complement, which facilitates immune attack against tumor. However, recent findings on tumor-promoting activities of complement suggest its dual action in cancer. The role of complement proteins in chronic inflammation, angiogenesis, immunosuppression and cancer cell signaling has been extensively reviewed [42,43]. Complement activation by tumor cells was observed in both *in vitro* studies and physiological fluids from cancer patients [44,45,46]. Moreover, significantly higher levels of complement components and activation fragments in plasma of LC patients were demonstrated [30,47,48]. High expression of complement regulatory proteins were associated with poor prognosis in colorectal and prostate tumors [49,50]. The results of our study should draw more attention to the possibility of use of complement-related proteins, especially C3, as new molecular cancer biomarkers. A more systematic analysis of abnormalities in the levels of complement components occurring in biofluids of cancer patients is needed, which can also contribute to better understanding of the dynamic interplay between cancer and complement.

The second protein, which was successfully identified as a NSCLC marker candidate in the current research is fibrinogen α chain (FIBA_HUMAN) protein. The peak of *m*/*z* 1545.6249, identified as a fragment of fibrinogen α chain, had a higher intensity in NSCLC patients compared to control subjects. Fibrinogen constitutes a major protein of human blood clotting and mediates platelet aggregation [51]. Fragments of fibrinogen α chain as well as fibrinopeptide A have been reported as markers of other tumor types, such as urothelial cancer [52], ovarian cancer [53], gastric cancer [54] and biliary tract cancer [32]. It was suggested that higher or lower intensities of various peptide ions in cancer can results not only from up- or downregulation of the parent proteins, but can originate from cancer-specific exoproteases as well. It was indicated that exopeptidase activities in particular overlapped with *ex vivo* coagulation can generate cancer-specific peptide fragments in serum [55,56]. Thus, those peptides may be treated as surrogate markers of cancer-derived proteases.

Since our results were obtained based on relative peptide intensities, new candidate protein biomarkers that are found using MALDI-TOF-MS analysis need verification and validation. For this purpose, two different quantitative analytical platforms: antibody-based technologies and mass spectrometry-based methods are used [57]. Since ELISA test did not show statistically significant differences in the expression of complement C3, further study will involve a quantitative approach to prove clinical utility of the other proteins from the proposed multi-component cancer signature.

## 4. Materials and Methods

### 4.1. Characteristics of the Study Participants

Serum samples were collected from ninety patients with a new diagnosis of LC, who were recruited in the Department of Thoracic Surgery, Poznan University of Medical Sciences. The criteria for the patient classification to the studied group were the following: diagnosis of NSCLC based on the histopathological examination of the biopsied or resected tissue specimens, there were no coexisting cancers, chemotherapy and/or radiotherapy and other cancer treatment were not used. Sixty-three healthy subjects, who underwent routine medical examination, without cancer and chronic metabolic diseases were enrolled to the control group. The control group was matched to the studied group in terms of age, sex and ethnic origin. Demographic and clinical profiles of the cancer patients and control individuals are presented in Table 1. The sera was collected in the same manner from LC patients and healthy subjects after an overnight fasting and stored –80 °C until analysis. The study was performed with full approval by the Local Ethical Committee of Poznan University of Medical Sciences, Poland (Decision No. 200/13) and in line with the Declaration of Helsinki. A written informed consent to use of serum samples in the study was received from all subjects.

### 4.2. Chemicals and Reagents

Ammonium acetate and trifluoroacetic acid (TFA) from Sigma Aldrich (St. Louis, MO, USA) were used. Ethanol, isopropanol, water, acetone and acetonitrile (ACN) were purchased from J.T. Baker (Center Valley, PA, USA). ClinProt standards, AnchorChip 800 µm target and α-cyano-4-hydroxycinnamic acid (HCCA) were from Bruker Daltonics (Bremen, Germany). Millipore Simplicity UV water purification system (Waters Corporation, Milford, MA, USA) was used to obtain deionized water. All used reagents were characterized by analytical grade or better.

### 4.3. Serum Samples Pretreatment

The solid phase extraction technique in the form of pipette tips pre-packed with hydrophobic phase was use as a sample pretreatment method. Prior MALDI-TOF-MS analyses, samples were diluted 1:5 using 0.1% TFA in water. In order to detect low molecular weight peptides depletion method using C18 ZipTip micropipette tips (Millipore, Bedford, MA, USA) was performed according to the manufacturer’s instruction. The protocol includes 3 steps: binding of proteins and peptides, washing, and elution. Fifty percent ACN solution was used for elution of the samples fractions. The detailed procedure was described in our previous study [41].

### 4.4. MALDI-TOF-MS Analysis

After preconcentration and purification 1 μL of each eluent sample was mixed with 10 μL of HCCA matrix solution (0.3 g/L HCCA in ethanol:acetone, 2:1, *v*/*v*), which was prepared daily. For the MS profiling analysis, 1 μL of the mixture was spotted directly onto the MALDI plate (AnchorChip, Bruker Daltonics, Bremen, Germany). Before analysis the spots were dried in the room temperature. MS analyses were performed using MALDI-TOF mass spectrometer, UltrafleXtreme (Bruker Daltonics, Bremen, Germany). The instrument was operated in positive-ion linear mode. Peak *m*/*z* values or intensities were recorded in the mass range of 1–10 kDa. The following ion source settings were used for MALDI-TOF MS analysis: ion source 1, 25.09 kV; ion source 2, 23.80 kV. Other parameters were set at: lens, 6.40 kV; pulsed ion extraction, 260 ns; and matrix suppression mass cut off, *m*/*z* 700 Da. Two thousand shots with a laser power of 80% were collected per one spectrum. Each sample was spread into 3 spots and analyzed in triplicate. External calibration of the mass spectrometer was proceeded with a mixture containing of Peptide Calibration Standard and Protein Calibration Standard I (1:5, *v*/*v*). The analyses were performed with average mass deviation less than 100 ppm. The three representative samples were analyzed on 3 different spots during a day and during 3 different days in order to evaluate the inter-day and intra-day reproducibility of MS. Eight randomly selected peaks with low, medium and high abundance peak area in the mass range of *m*/*z* 1–10 kDa were marked for variability assessment. Calculation of CV values allowed to measure the variability of each MS peak area. For data collection and instrument control FlexControl 3.4 software (Bruker Daltonics, Bremen, Germany) was applied. The software allowed for automatic acquisition and processing of the obtained spectra.

### 4.5. LC-MS/MS Identification of Selected Ion Peptides

The sample was pretreated with C18 ZipTip micropipette tip. The obtained eluent was concentrated and separated using nano-liquid chromatography (EASY-nLC II, Bruker Daltonics). The set was consist of the trap column (NS-MP-10 BioSphere C18, NanoSeparations, Nieuwkoop, the Netherlands; 20 mm × 100 μm I.D., particle size 5 μm, pore size 120 Å) and column (Thermo Scientific Acclaim PepMap 100 C18, Thermo Scientific, Sunnyvale, CA, USA ; 150 mm × 75 μm I.D., particle size 3 μm, pore size 100 Å). The separation was performed by linear gradient of 2%–50% of ACN for 150 min (0.05% TFA water solution—mobile phase A, and 0.05% TFA 90% ACN—mobile phase B). The flow rate was 300 nL/min. The 2 µL of the sample was injected on the column. Sample collection was started in the 54 min of the running gradient. From this, 384 sample fractions directly mixed with matrix solution were automatically spotted onto an AnchorChip™ target with time intervals of 15 s by using fraction collector (Proteineer-fc II, Bruker Daltonics, Bremen, Germany). Matrix solution contained: 748 μL of mixture of acetonitrile and 0.1% TFA (95:5 *v*/*v*), 36 μL of HCCA saturated solution of 0.1% TFA and acetonitrile (90:10 *v*/*v*), 8 μL of 10% TFA and 8 μL of 100 mM ammonium phosphate monobasic. For control of the nano-LC system HyStar 3.2 software (Bruker Daltonics) was used. The obtained fractions were further analyzed using the MALDI-TOF/TOF instrument (UltrafleXtreme, Bruker Daltonics,). The Peptide Calibration Standard mixture (Bruker Daltonics) was used for the external calibration. For the MS-analysis the software WARP-LC (Bruker Daltonics,) was used to establish a list of precursor ions. The chosen *m*/*z* (1520.8456, 1545.6249, 1568.67, and 1616.67) were subjected to MS/MS fragmentation mode. The identification of the proteins was conducted with settings for MS and MS/MS mode as previously described [41]. FlexControl 3.4, FlexAnalysis 3.4 and BioTools 3.2 (Bruker Daltonics) were used for the control of the mass spectrometer, acquisition, processing and evaluation of the data. The SwissProt database with Mascot 2.4.1 search engine (Matrix Science, London, UK) was used to analyze the results. Only searches restricted to “*Homo sapiens*” were taken into account. The peptide precursor mass tolerance was 50 ppm, while fragment mass tolerance 0.7 Da. Other search parameters for protein identification were as follows: monoisotopic mass, no enzyme used, peptide charge: +1.

### 4.6. Determination of Complement C3 by ELISA

A sandwich enzyme immunoassay (ELISA) kit was used for the quantitative measurement of complement C3. Assays were carried out as described in the manufacturer’s manual (Cloud-Clone Corp., Houston, TX, USA). The test was validated for in vitro quantitative measurement of C3 in human serum within detection range 7.8–500 ng/mL.

### 4.7. Data Analysis

Each spectrum was analyzed using ClinProTools software (version 3.0; Bruker Daltonics). As each sample was measured in triplicate, the spectra grouping function was used to average the obtained data. Then, the spectra were processed with the following manner: normalization to the total ion current (TIC), recalibration using the prominent common *m*/*z* values, baseline “top hat” subtraction with the minimum baseline width set to 10%, smoothing, the signal-to-noise ratio ≥5, peak picking and peak calculation operation. Spectra were processed in the 1–10 kDa range. Peak intensity differences between the analyzed groups were evaluated using either a student t-test or Wilcoxon test (depending on variable distribution). In all tests, *p* ≤ 0.05 was considered to indicate a statistically significant difference. Receiver operating characteristic (ROC) curve was calculated for each peak to show a graphical overview between sensitivity and specificity. The area under the ROC curve (AUC) enabled to compare discriminatory abilities of the serum peptide ions. In order to obtain discriminating models, 3 algorithms were used: genetic algorithm (GA), supervised neural network (SNN) and quick classifier (QC). The algorithms were used to integrate the best number of peaks in discrimination models. Determination of a common signature among the obtained spectra of each of the studied groups lead to proper classification of the test samples by the model [58]. GA imitates evolution in nature and selects a combination of variables that are the most significant for the class separation, while less capable are abandoned. The optimal class separation with high variance between classes is obtained by optimizing a cost function [59,60]. QC represents a univariate sorting algorithm. Generation of a QC model is based on storing the class averages of the peak areas together with p-values obtained from a *t*-test. First, the peak areas or intensities are sorted per peak and next a weighted average from all peaks need to be calculated [59,61]. SNN algorithm uses a prototype-based classification. The characteristic spectra of each study class, which are named prototypes, are identified. Then, they are considered as prototypical samples of that class from a proteomic point of view [59,61]. Two indicators of the model’s performance, 20% leave one out cross validation (10 iterations) and recognition capability, were calculated for all three used algorithms. The cross validation values indicate the reliability of the generated model, whereas the recognition capability values show the model’s capability of correct identification of the component spectra [62]. Sixty-seven samples from LC patients (29 adenocarcinomas and 38 squamous cell carcinomas) and 47 healthy control samples were randomly selected to a training set (Table 1). The rest of the samples, 23 LC samples (11 adenocarcinomas and 12 squamous cell carcinomas ) and 16 control samples, were used as a test set. The aim of using the training set to build the model was to define spectra of the model classes in such a way that new spectra (the test set) might be classified afterwards. This external validation procedure was applied to the model of the best performance and enabled to calculate sensitivity and specificity values. Peptides of the highest discriminatory ability were subjected to further experiments with the aim of their identification as fragments of specific proteins. The validation data concerning differential expression of identified proteins was analyzed using univariate statistical analyses by applying Statistica software (version 12.0; StatSoft Inc., Tulsa, OK, USA).

## 5. Conclusions

We used proteomic profiling strategy in searching of serum peptide signatures of NSCLC. The combination of the ZipTip technology with MALDI-TOF-MS for the analysis of serum peptide pattern of NSCLC patients was presented for the first time. Based on the observed significant differences between cancer patients and control subjects, the classification model was generated, which allowed for accurate group discrimination. The model turned out to be robust enough to discriminate new validation set of samples with satisfactory sensitivity and specificity. Two peptides from the diagnostic pattern for NSCLC were identified as fragments of C3 and fibrinogen α chain. There is a risk that the identified peptide ions may not be specific for NSCLC, but also represent tumor indicators in general. Therefore, further attempts are planned to identify other components of the developed classification model, which can be more specific for NSCLC. Since ELISA test did not confirm significant differences in the expression of complement component C3, further study will involve a quantitative approach to prove clinical utility of the other proteins from the proposed multi-peptide cancer signature.

## Figures and Tables

**Table 1:** Characteristics of non-small cell lung cancer (NSCLC) patients and control subjects.

Characteristic	Training Set		Test Set		Total	
	NSCLC Patients	Controls	NSCLC Patients	Controls	NSCLC Patients	Controls
**No. of Subjects**	67	47	23	16	90	63
Sex						
Male	40 (59.7%)	31 (66.0%)	18 (78.3%)	10 (62.5%)	58 (64.4%)	41 (65.1%)
Female	27 (40.3%)	16 (34.0%)	5 (21.7)	6 (37.5%)	32 (35.6%)	22 (34.9%)
Age						
Mean (SD)	63 (6.5)	62 (8.5)	66 (8.0)	62 (10.0)	64 (6.9)	62 (8.7)
Range	48–86	43–78	53–81	45–77	48–86	43–78
Histological type						
Squamous cell carcinoma	39 (58.2%)		12 (52.2%)		50 (55.6%)	
Adenocarcinoma	28 (41.8%)		11 (47.8%)		40 (44.4%)	
Grade of cancer differentiation						
G1	1		1		2	
G2	31		12		43	
G2/3	6		2		8	
G3	23		6		29	
Unknown	6		2		8	
TNM stage						
IA	14		3		17	
IB	19		6		25	
IIA	14		5		19	
IIB	8		2		10	
IIIA	12		7		19	

**Table 2:** Intensities (mean ± SD) and results of the univariate statistical analyses of the most discriminating peptide ions determined in serum of non-small cell lung cancer patients (NSCLC) (*n* = 90) and control group (*n* = 63).

Mass (*m*/*z*)	NSCLC	Control Group	*p*-Value ^1^	AUC ^2^
1568.45	6.33 ± 4.27	2.75 ± 0.91	<0.000001	0.85
1546.72	54.19 ± 41.34	24.09 ± 14.03	0.00000386	0.78
1617.88	66.99 ± 48.77	29.24 ± 23.84	0.00000386	0.78
1520.16	13.56 ± 4.36	9.49 ± 4.28	0.00003860	0.78
4466.98	0.31 ± 0.19	0.51 ± 0.23	0.00002280	0.75
4803.17	0.29 ± 0.18	0.56 ± 0.34	0.00000975	0.77
4787.36	0.52 ± 0.48	1.21 ± 1.12	0.00001960	0.79
2083.30	1.07 ± 1.42	2.24 ± 2.57	0.00001960	0.76

^1^ Calculated based on *t*-test or Wilcoxon test. ^2^ Area under the ROC curve.

**Table 3:** List of peptide ions included to the generated classification models and diagnostic performances of the models.

	Non-Small Cell Lung Cancer ^1^			Lung Adenocarcinoma			Lung Squamous Cell Carcinoma		
	Quick Classifier	Supervised Neural Network	Genetic Algorithm	Quick Classifier	Supervised Neural Network	Genetic Algorithm	Quick Classifier	Supervised Neural Network	Genetic Algorithm
Mass [Da]	1520.16	1546.72	1466.90	1568.45	1568.45	1450.94	1520.16	1546.72	2884.63
	1538.14	1617.88	1568.45	7923.94	1305.41	4626.52	1617.88		1527.52
	1546.72	1505.34	1450.94		5004.12	1546.72	1741.57		1520.16
	1568.45		1880.97		2755.6	1505.42	2884.63		1564.1
	1617.88		1617.88		2604.28	6630.86	4787.36		1546.72
	4466.98		1520.16		1450.94	1466.85			5808.48
	4680.61		1546.72		1628.01	9289.98			3882.95
	4803.17		5904.39		1741.49	1510.24			6589.21
	6528.67		6330.86		2673.89	6304.32			1078.16
			6432.69		2555.04	1021.00			5919.08
					1905.19				
					4787.36				
					2575.89				
					1888.66				
					1546.72				
Cross validation (%) ^2^	75.54	47.87	71.89	84.31	86.35	74.71	69.51	60.64	74.66
Recognition capability (%) ^2^	76.33	56.47	96.22	87.46	95.79	97.87	75.80	50.86	96.51
Specificity (%) ^3^			64.40		66.70		-	-	86.70
Sensitivity (%) ^3^			94.40		72.70		-	-	66.70

^1^ Without division into histological types. ^2^ Calculated using a training set. ^3^ Calculated using a test set.

## Supplementary Materials

Supplementary materials can be found at http://www.mdpi.com/1422-0067/17/4/410/s1.

## Abbreviations

MALDI-TOF-MS: Matrix-assisted laser desorption/ionization time-of-flight mass spectrometry
LC: Lung cancer
NSCLC: Non-small cell lung cancer
ROC curve: Receiver operating characteristic curve
AUC: Area under the ROC curve
GA: Genetic algorithms
QC: Quick classifier
SNN: Supervised neural network
TFA: Trifluoroacetic acid
ACN: Acetonitrile
HCCA: α-Cyano-4-hydroxycinnamic acid
