# Supplementary material for: Identification of Serum Peptidome Signatures of Non-Small Cell Lung Cancer
Source: Int J Mol Sci. 2016 Mar 31;17(4):410. doi: 10.3390/ijms17040410 (PMC4848884; doi:10.3390/ijms17040410)
Supplement: Supplementary file 1 [file ijms-17-00410-s001.pdf]

Supplementary Materials: Identification of Serum Peptidome Signatures of Non-Small Cell Lung Cancer

Agnieszka Klupczynska, Agata Swiatly, Joanna Hajduk, Jan Matysiak, Wojciech Dyszkiewicz, Krystian Pawlak and Zenon J. Kokot

Table S1. Intra-day reproducibility.

m/*z* (Da)	Peak Area			CV (%)
	Spot 1	Spot 2	Spot 3	
1,866.78	54.85	49.96	57.06	6.73
1,451.04	28.87	26.86	30.03	5.61
1,207.54	65.43	54.82	65.64	9.99
2,555.19	3.65	3.53	3.68	2.19
1,546.84	26.62	22.61	27.27	9.9
4,210.23	6.67	6.12	6.23	4.59
3,263.78	5.09	4.47	4.96	6.8
2,933.5	7.37	6.23	7.42	9.61
	Average CV %			6.92

Table S2. Inter-day reproducibility.

m/*z* (Da)	Peak Area			CV (%)
	Day 1	Day 2	Day 3	
1,351.74	69.34	43.97	46.36	26.32
1,467.05	339.53	332.77	349.17	2.42
1,546.91	25.5	23.45	24.6	4.19
1,618.09	66.04	65.48	68.47	2.38
3,192.86	3.37	8.52	6.79	42.09
2,933.69	7.01	11.68	15.53	37.40
2,770.56	3.51	6.14	5.8	27.78
4,210.32	6.34	6.19	8.86	21.04
	Average CV %			20.45

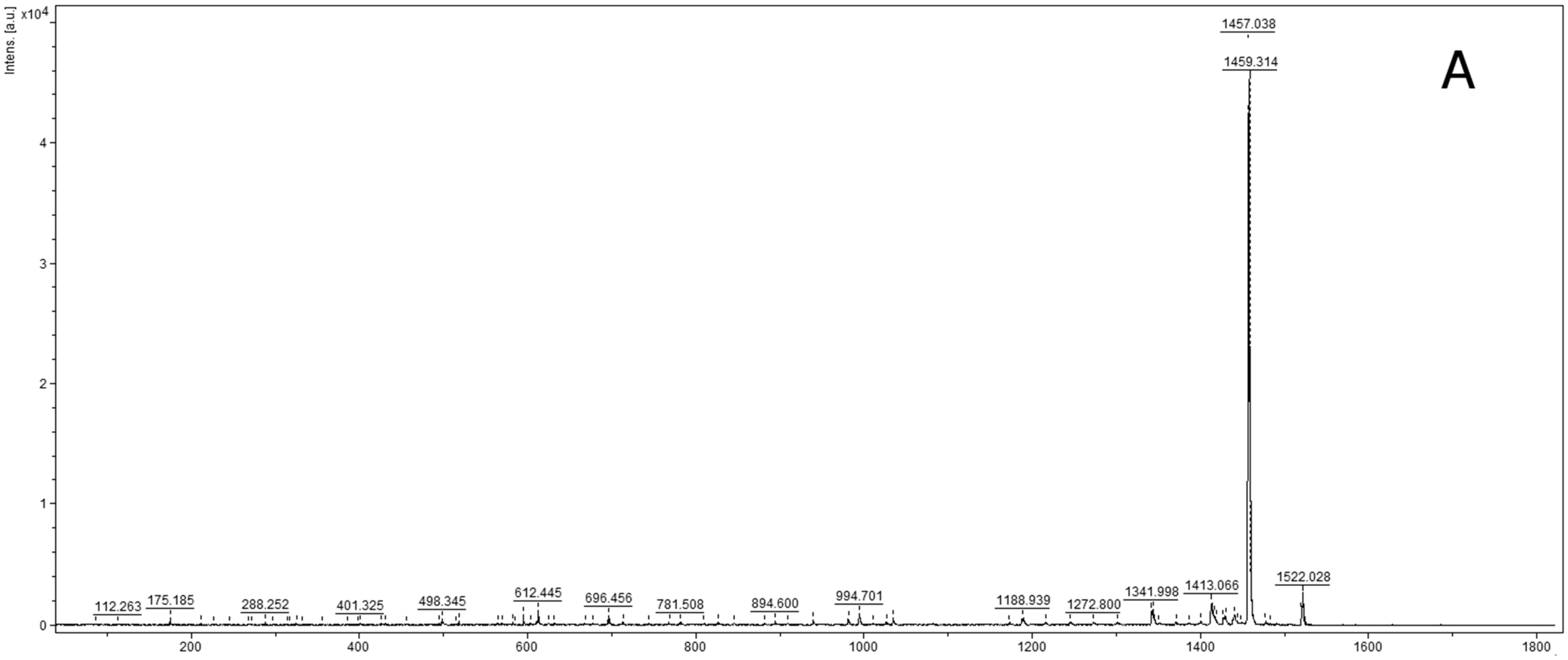

Figure S1. *Cont.*

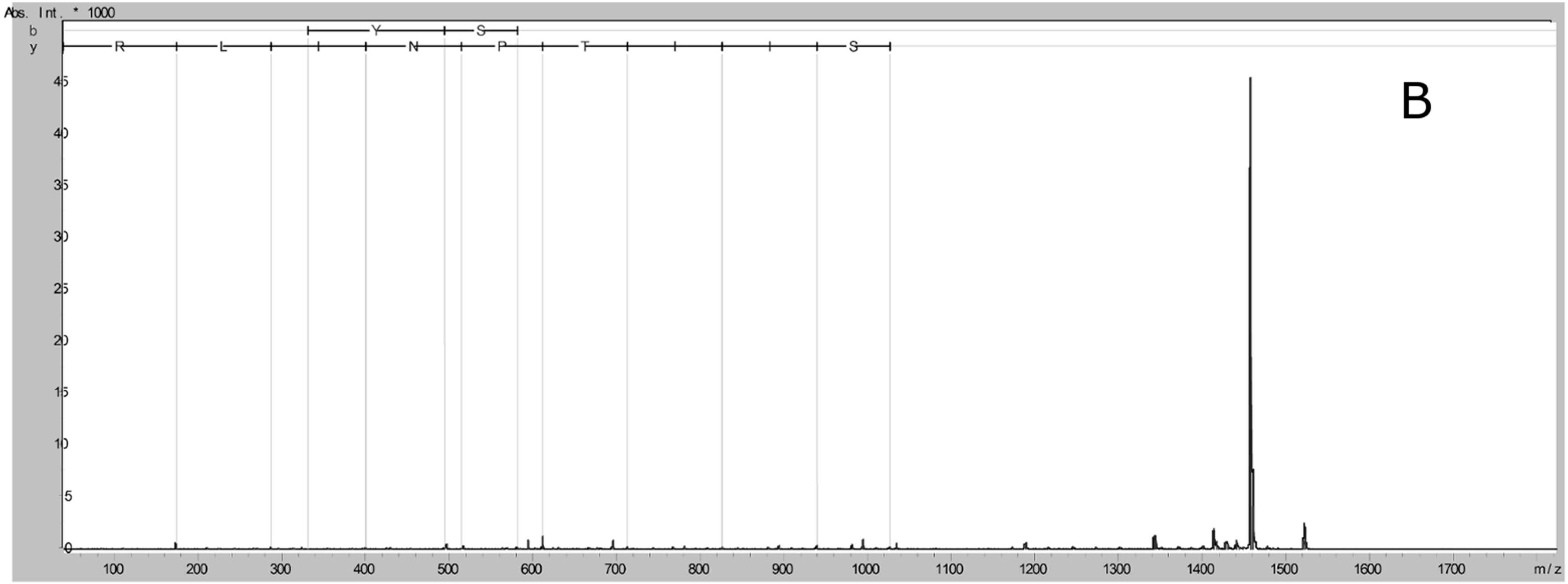

Figure S1. MS/MS spectrum of peptide ion *m*/*z* 1520.8456 Da (**A**); and MS/MS spectrum of peptide ion *m*/*z* 1520.8456Da with assigned amino acid sequence (**B**).

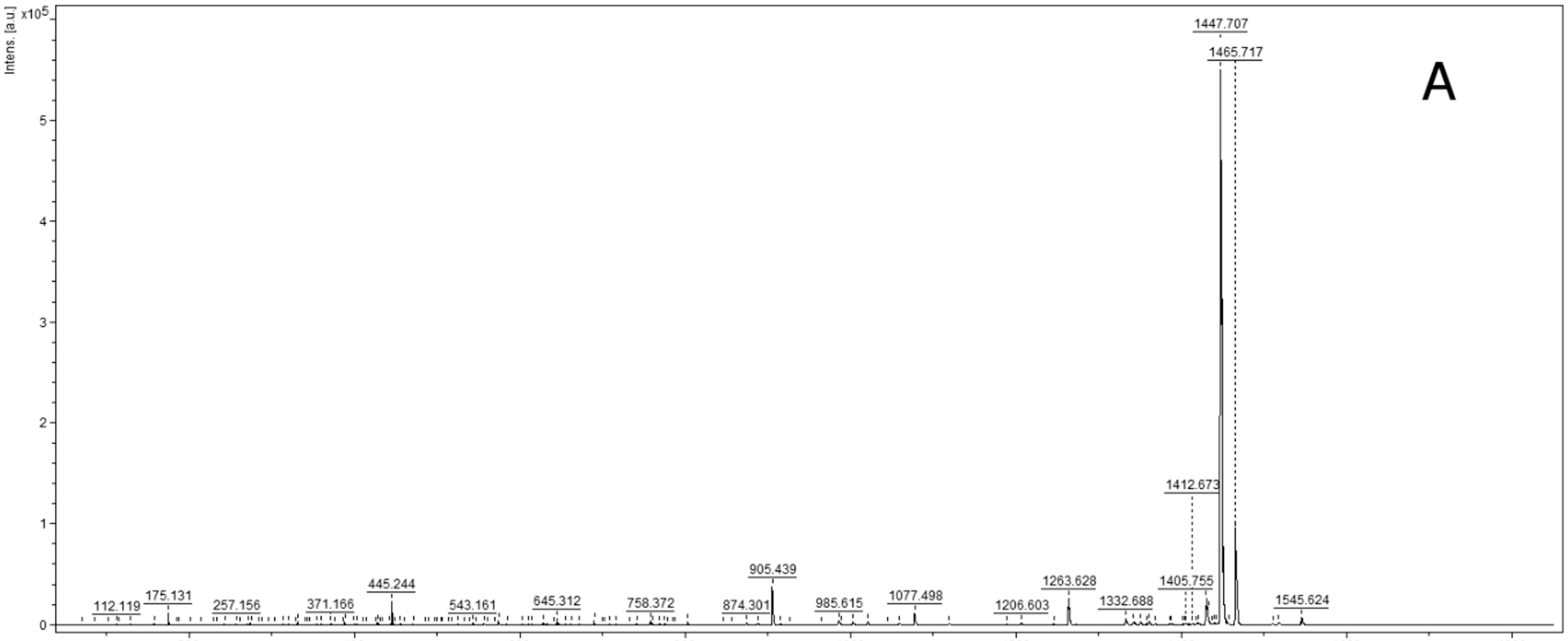

Figure S2. *Cont*.

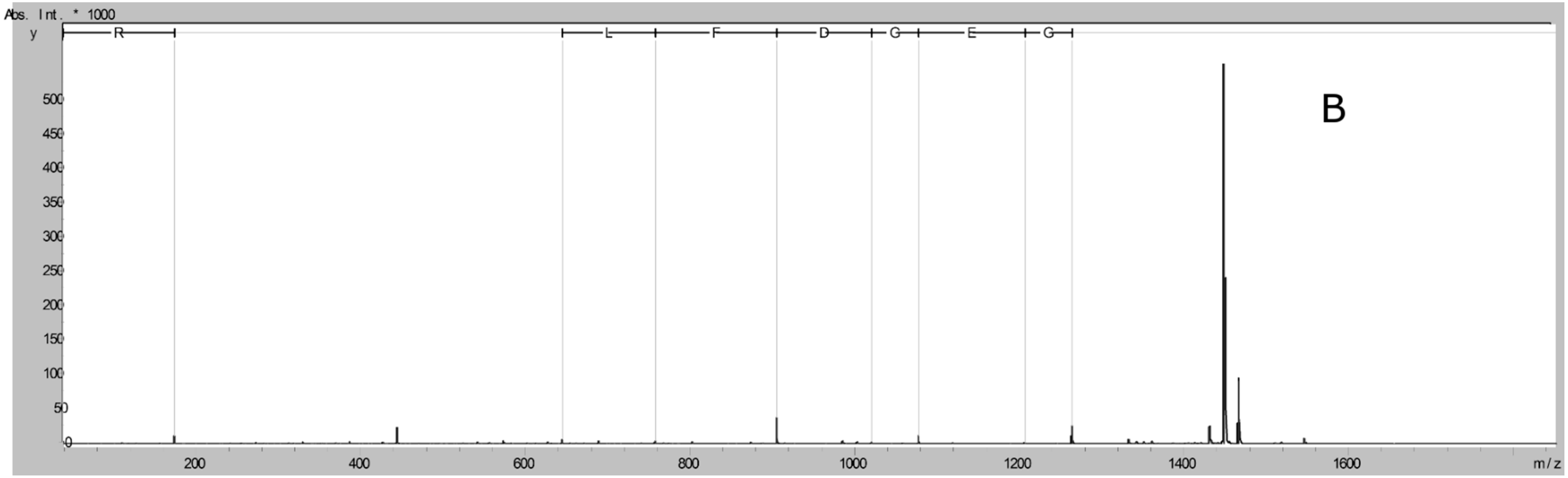

Figure S2. MS/MS spectrum of peptide ion *m*/*z* 1545.6249 Da (**A**); and MS/MS spectrum of peptide ion *m*/*z* 1545.6249 Da with assigned amino acid sequence (**B**).
